# Stability of Non-Concentric, Multilayer, and Fully Aligned Porous MoS_2_ Nanotubes

**DOI:** 10.3390/membranes12080818

**Published:** 2022-08-22

**Authors:** Pablo Jahir Peña-Obeso, Rafael Huirache-Acuña, Fernando Iguazú Ramirez-Zavaleta, José Luis Rivera

**Affiliations:** 1 Facultad de Ingeniería Química, Universidad Michoacana de San Nicolás de Hidalgo, Morelia 58000, Mexico; 2 Facultad de Ciencias Físico–Matemáticas, Universidad Michoacana de San Nicolás de Hidalgo, Morelia 58000, Mexico

**Keywords:** MoS_2_, nanotube, multilayer, polytype, ring-like

## Abstract

Nanotubes made of non-concentric and multiple small layers of porous MoS_2_ contain inner pores suitable for membrane applications. In this study, molecular dynamics simulations using reactive potentials were employed to estimate the stability of the nanotubes and how their stability compares to macroscopic single- (1L) and double-layer MoS_2_ flakes. The observed stability was explained in terms of several analyses that focused on the size of the area of full-covered layers, number of layers, polytype, and size of the holes in the 1L flakes. The reactive potential used in this work reproduced experimental results that have been previously reported, including the small dependency of the stability on the polytype, the formation of S–S bonds between inter- and intra-planes, and the limit of stability for two concentric rings forming a single ring-like flake.

## 1. Introduction

MoS_2_ is a metal dichalcogenide used in a variety of technological applications: catalysis [1,2,3], optoelectronics [4,5,6], tribology [7,8,9], etc. Three polytypes of MoS_2_ have been extensively studied and reported in the literature: 1T, 2H, and 3R, where the numbers indicate how many layers are needed to fully describe its unit cell, with the 2H polytype being the most thermodynamically stable [10,11,12]. In the 2H MoS_2_ polytype, the surfaces of two layers interact via the external S atoms and have flipped orientations and dipoles. In the 3R MoS_2_ polytype, the orientation of the three layers and their dipoles are the same, but they do not form a perfect stack; instead, the individual layers are displaced laterally to minimize the separation between the layers [11]. Monolayer (1L) MoS_2_ is commonly obtained through mechanical exfoliation [13,14], whereas Chemical Vapor Deposition (CVD) is often used to produce multilayer (ML) MoS_2_ [15,16], and CVD on confined spaces can produce crystalline ML MoS_2_ [17,18]. ML MoS_2_ flakes exhibit improved properties that are useful in electronic and optoelectronic devices due to their higher density of states [18,19,20]. In catalytic reactions, ML MoS_2_ flakes strained on Au surfaces showed some improved properties for the hydrogen evolution reaction, comparable to Pt surfaces [21].

Membranes in 2D materials can be sculpted with precise spatial control and flexibility of the required pattern design [22,23,24]. The pores in these membranes are created by electron beam irradiation, photothermal methods, high temperatures, or ion bombardment, which create defects that can form the pores of the membranes after they have healed. The defects are the result of several processes such as the “knock-on” effect, ionization, or chemical etching induced by the electron beam. A typical defect in MoS_2_ flakes is the vacancy of a single or multiple S atoms. Theoretical calculations have estimated the threshold energy required to displace S atoms in pristine MoS_2_ as ~6.5 eV, in agreement with experimental results using electron beams [25,26].

Nanofiltration of organic solvents can be achieved using MoS_2_ flakes with a few layers, aligned to form a membrane and filter the solvent from solutes larger than 1 nm [27]. Such filtration is effectively realized between the borders of the flakes that once aligned, interacting at close distances (less than 1 nm), allowing the solvent to cross the space formed by the border–border separation that occurs, while blocking larger molecules of solute. Porous MoS_2_ flakes can be obtained by exfoliation processes to produce flakes with a few layers, which are subjected to cavitation forces to make holes in the flakes. Many of these flakes are partially aligned and allow the permeation of solvent molecules while blocking atoms or molecules of solute larger than the size of the pores. Porous MoS_2_ partially aligned with large pore sizes (between 10 and 50 nm) can reject between three and four times more NaCl than partially aligned full-covered flakes, when used to desalinate a 0.5 M solution [28]. Covalent functionalization of MoS_2_ membranes with acetic acid can improve the rejection percent (up to 92%) of Na_2_SO_4_ [29]. 

In this work, we used reactive molecular dynamics and studied the possible formation of MoS_2_ nanotubes based on fully aligned 1L MoS_2_ flakes with holes that have smaller diameters than those used to filtrate solutions [28]. Previously reported MoS_2_ nanotubes have been made of a single, large MoS_2_ layer, bent, and connected through their borders [30], while the proposed nanotubes in this work are made of multiple 1L MoS_2_ porous flakes, repeated along the normal direction of the nanotube. We estimated how stable, finite, or periodic multiple 1L MoS_2_ nanotubes are under vacuum conditions. We explained the stability of the nanotubes in terms of their polytype and the properties of their constituent 1L porous MoS_2_. The studied properties were based on the effects on the stability due to different factors such as the area of the MoS_2_ flakes, the size of the hole at the center of the flake, the number of layers in the nanotube, and its polytype. Future work will focus on the stability of the proposed nanotubes under aqueous conditions and the use of these nanotubes in membrane setups useful for separating fluid of interest.

## 2. Methods

We used the Molecular Dynamics methodology with a reactive force field (REAXFF) [31,32] to study 1L, double layers (2L), and ML full-covered and porous flakes of MoS_2_ resulting in hexagonal structures, similar to those proposed by Lauritsen et al. [33], with sizes ranging from Mo_12_S_24_ to Mo_243_S_486_. Larger flakes could not be simulated due to prohibitive computational resources, especially considering the large periods of simulation required to obtain stable configurations. The conformation of each full-covered flake was built as concentric and contiguous rings of MoS_2_ units. Hollow flakes were built using the equilibrated conformation of full-covered flakes and eliminating an increasing number of central rings of MoS_2_ units to simulate holes with several inner diameters and a constant outer diameter. The syntax for naming the porous flakes was based on combining the formula of the full-covered flake with the formula corresponding to the eliminated central flake, separated by a hyphen. Full-covered and hollow ML flakes were built by replicating equilibrated conformations of 1L, using orientations corresponding to 2H or 3R polytypes of MoS_2_. The dynamics of the system evolved under vacuum conditions using the Berendsen thermostat, which kept the temperature, T, of the flakes around a constant value, with the number of atoms and the simulation cell volume invariable (NVT) [34]. Some of the systems were also simulated without any thermostat (constant total energy) but with the rest of the variables kept constant (NVE); the initial configurations for these simulations used equilibrated positions and momentums from simulations under NVT conditions. The linear and angular momentums were removed from the systems to eliminate any undesired effects on the flake stability. The time step used was 0.1 fs, which maintained the stability of the convergence of the point charges of the atoms. The interaction between the electronic charges of the atoms was calculated using the particle-particle particle-mesh network (PPPM) algorithm [35]. The algorithms were implemented in the open-source Large-Scale Atomic/Molecular Mass Parallel Simulator (LAMMPS) [36].

The reactive potential for the interaction between the atoms was developed by Ostadhossein et al. [37], which was optimized to study properties such as the formation energies of five different types of vacancies on MoS_2_, various barriers to the migration of vacancies, and the barrier to the transition between the 2H semiconductor polytype and the 1T metallic polytype. This interaction potential has also been used to study the mechanisms of MoS_2_ formation from molybdenum trioxide sulfurization [38]. Chen et al. also used this potential to study the stoichiometric effect of oxygen on the formation of crystalline MoS_2_ from its amorphous state [39]. Furthermore, Noori et al. used this potential to study the formation of nanopores in 1L MoS_2_ subjected to nanoparticle impacts [40].

The initial conformation of the Mo_12_S_24_ flake, which was used to model the initial conformation (bond distances and angles) of the rest of the flakes, corresponded to the conformation of the minimum energy at 0 K, which used the Density Functional Theory (DFT), the hybrid exchange functional–correlation B3P86, and the effective interaction potentials of LANL1 (Mo) and LAN2 (S), previously carried out by Zakharov et al. [41].

## 3. Results and Discussions

### 3.1. Full-Covered 1L Flakes

**Figure 1:**
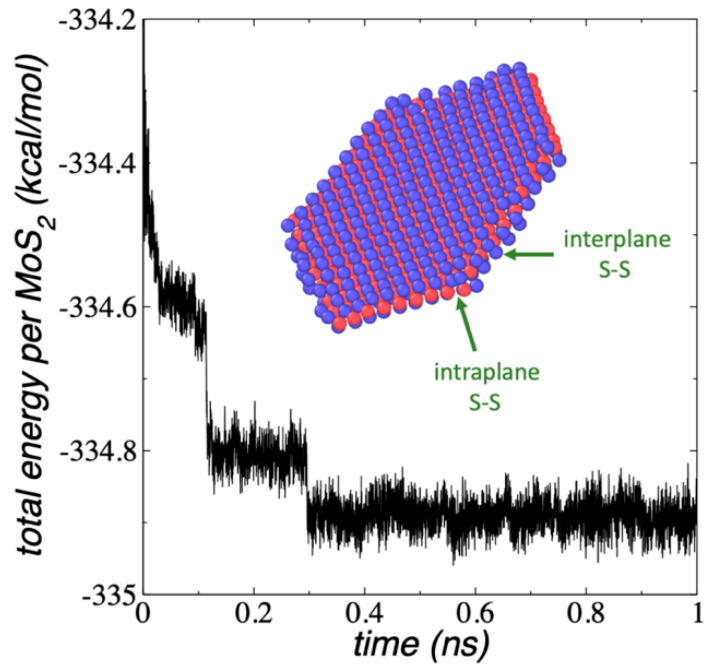
E_#MoS2_ of 1L Mo_243_S_486_ flake as a function of simulation time, under vacuum and NVT conditions at 298.15 K. The inset snapshot corresponds to the equilibrated conformation of the flake and shows the formation of intra- and inter-plane S–S bonds. Blue and red spheres represent Mo and S atoms, respectively.

The properties of complex and unseen nanoparticles, such as those proposed in this work, can be understood in terms of the properties of simpler nanoparticles with similar nature. To explain the properties of complex structures, such as layered nanotubes of porous MoS_2_ flakes, we first studied the mechanical properties of their simpler constitutive structures, the 1L MoS_2_ flakes, full-covered and porous. We simulated flake structures ranging from Mo_12_S_24_ to Mo_243_S_486_ using REAXFF under vacuum and NVT conditions at 298.15 K. The smallest flake (Mo_12_S_24_) contained a central ring with three MoS_2_ units and an outer ring with nine MoS_2_ units. The next flake (Mo_27_S_54_) was based on the conformation of the two central rings of Mo_12_S_24_ and a third outer ring with fifteen MoS_2_ units. The rest of the larger flakes were based on the conformation of the smaller flakes and contained subsequent additional rings of MoS_2_ units. All the flakes formed a hexagonal structure with two border types: three borders exposing bare Mo atoms and three borders exposing bare S atoms. In triangular MoS_2_ flakes, only one type of border is possible, but in the proposed hexagonal shape, both low-index borders are possible, and even the bare Mo borders can be stabilized further if they are partially covered with S atoms [33,42]. In this work, we did not consider additional stabilizations of the Mo borders because there were no interactions between the borders with other structures. The diameters of the flakes were in the range of 0.86 nm (Mo_12_S_24_) to 4.78 nm (Mo_243_S_486_). The dynamics of the evolution of the conformation of the 1L Mo_243_S_486_ flake is shown in Figure 1 in terms of the total energy of the system divided by the number of MoS_2_ units, E_#MoS2_. It takes about 300 ps for the Mo_243_S_486_ flake to reach the equilibrium conformation at 298.15 K, and from there, its energy oscillated around a constant value. Smaller flakes needed less time to reach the equilibrium conformation at the same T, i.e., the equilibration of Mo_12_S_24_ flake took only a few ps.

The downhill behavior of the E_#MoS2_, observed in Figure 1 for the Mo_243_S_486_ nanoparticle, corresponded to chemical changes within the nanoparticle, which produced the formation of S–S bonds at the edges of the flake (inset of Figure 1). The S–S bond formation did not show energetic barriers in the total energy profiles (Figure 1) nor the potential energy profiles (not shown). Similar energetic changes have been observed in downhill (barrierless) protein folding [43,44]. The types of S–S bonds formed are shown in the inset of Figure 1. The 1L MoS_2_ are formed by a central plane of Mo atoms bonded to two outer planes of S atoms. The S–S bonds can form between two different planes of S atoms (interplane) and are the most common. The least frequent S–S bonds are those that occur in the same plane (intraplane). The S–S bonds at the layer edges have been previously reported; they stabilize the nanoparticle edges, and in sufficient quantity, they can change the magnetic state of the nanoparticle [45,46]. S–S bond formation is also induced when layers of MoS_2_ are exposed to mechanical loads in the range of GPa [47]. The formation of S–S bonds was present in all the studied nanoparticles. The analysis of the energy profiles of the flakes does not show a unique value in the energy drop for the formation of each type of S–S bond, which suggests that their formation occurs through several mechanisms.

**Figure 2:**
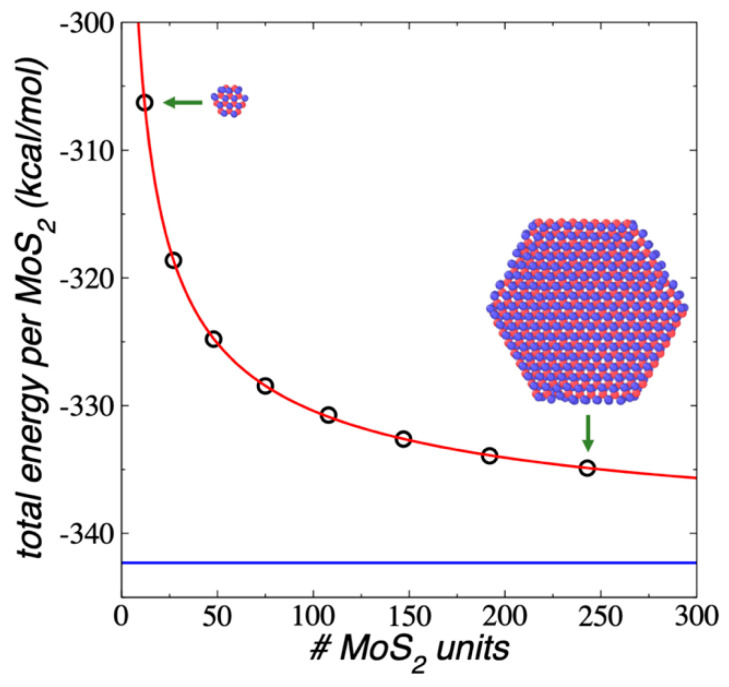
E_#MoS2_ of 1L flakes (black circles) as a function of the number of MoS_2_ units, under vacuum and NVT conditions at 298.15 K. The red line represents a fitting to an exponential decay expression and the blue line represents the plateau value. The inset snapshots correspond to the equilibrated conformations of the smallest (Mo_12_S_24_) and largest (Mo_243_S_486_) flakes. Blue and red spheres represent Mo and S atoms, respectively.

The E_#MoS2_ in each flake at 298.15 K were calculated and are shown in Figure 2 as a function of the number of MoS_2_ units in the flake. The E_#MoS2_ showed an exponential growth with the increasing number of MoS_2_ units in the flake. The difference between the convergence value at a very large number of MoS_2_ units differed from the value of the largest flake studied in this work (Mo_243_S_486_) by about 7.41 kcal/mol (0.32 eV), which means that on average the cohesivity of each MoS_2_ unit in this flake is 0.32 eV less cohesive (less stable) than those present in flakes with a much larger area (macroscopic flakes). The smallest flake (Mo_12_S_24_) showed differences as large as 36.03 kcal/mol (1.56 eV) compared with the macroscopic value. Extrapolations using an exponential decay curve fitted to the data predicted that nanoparticles like Mo_2,187_S_4,374_ are required to have flakes that are 0.1 eV less stable than those that would occur in macroscopic flakes, and a flake like Mo_155,552_S_311,104_ is needed to have differences of 0.01 eV. These large flakes will have diameters of 29 and 80 nm, respectively, and these diameters are six and twenty times larger than the diameter of the largest flake studied in this work, respectively. Very large systems, with sizes in the scale of hundreds of nm, are also needed to reproduce macroscopic uncertainties in other properties, as the critical thickness and surface tension of atomic and polymeric fluids under vapor/liquid or vacuum/liquid equilibrium [48,49,50].

**Figure 3:**
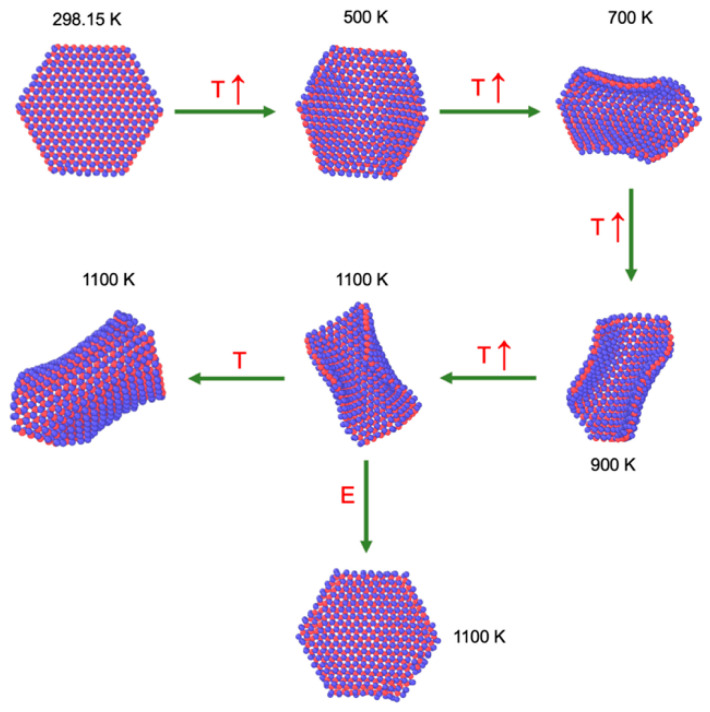
Snapshots of the 1L Mo_243_S_486_ flake under vacuum. From 298.15 to 1100 K, the T of the flake was increased at a rate of 100 K/300 ps. At 1100 K, the flake was maintained at constant T (NVT) for several ns. In a parallel simulation, the flake was also simulated under NVE conditions for several ns. Blue and red spheres represent Mo and S atoms, respectively.

**Figure 4:**
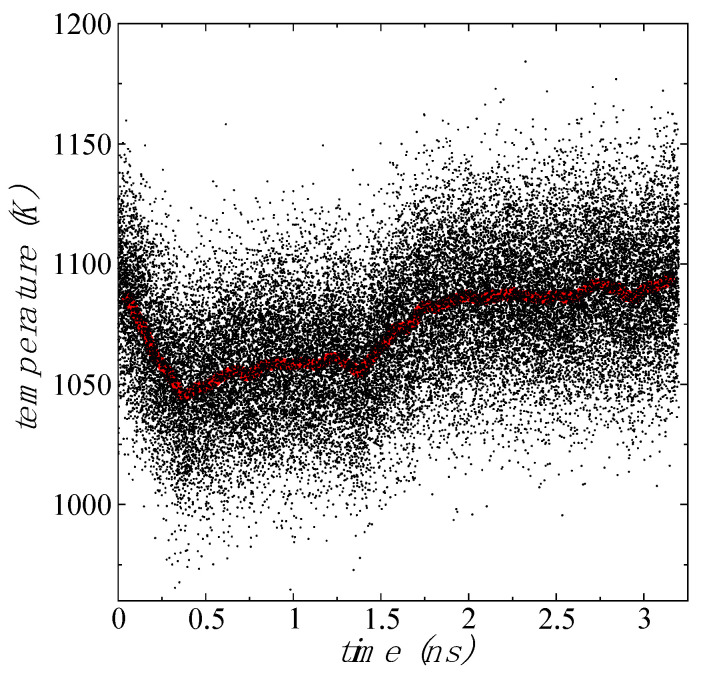
Temperature of 1L Mo_243_S_486_ flake as a function of the simulation time, under vacuum and NVE conditions starting from an equilibrated configuration at 1100 K (NVT). Points represent time averages for every 100 configurations. Red line represents the average value for every 1000 configurations.

We studied the effects of T on the conformational changes of 1L MoS_2_ flakes and increased the T linearly at a rate of 100 K/300 ps. At the beginning, the additional kinetic energy increased the in-plane vibrations, but between 300 and 400 K, the layers started to bend and unbend periodically. At 1100 K, close to the experimental limit of thermal stability for ultrathin MoS_2_ layers [51], the bending behavior was more pronounced. We maintained the T at 1100 K (NVT) and an animation of the bending behavior is shown in the Supplemental Information (Video S1), which can be described as a periodic bending and unbending of the flake with opposite borders almost contacting. The bending borders changed periodically, taking turns, and forming a central cavity at the center of the flake, like the process of making lemonade and squishing a lemon by hand. In Figure 3, we showed snapshots of typical conformations at T between 298.15 and 1100 K. We maintained the T at 1100 K for several ns, and the periodic bending also maintained its behavior. To verify what induces the bending behavior, we simulated the flake under NVE conditions, starting from the last stable configuration (positions and momentums) at 1100 K. After a couple of ns, the flake stopped bending and recovered the in-plane vibration behavior. Initially, during the NVE simulation, the T of the flake dropped 50 K in 0.4 ns, and after that, it slowly increased to reach the original T of 1100 K (Figure 4). Under NVE conditions, the thermostat stops an induced behavior at the borders, and the additional kinetic energy at the borders (gained during the process under NVT conditions) is transformed into potential energy, probably contributing to more cohesive in-plane interactions, which slowly is transformed again into in-plane kinetic energy until the system recovered its original T. Therefore, the observed bending behavior of flexible 1L flakes at NVT conditions is an artifact created by the thermostat. The continuous application of a thermostat on the flexible 1L flake, induced a cumulative behavior of kinetic energy at the borders, which induces the bending process, and once the bending reaches a maximum deformation of the flake, restoring forces at the center of the flake retracts the bent borders. The recovery of the T under NVE conditions indicated that the value of the total energy is the same in the bending and non-bending layers and the difference between both states (bending and non-bending) probably originates from how the total energy is distributed between its contributions (kinetic and potential energy).

**Figure 5:**
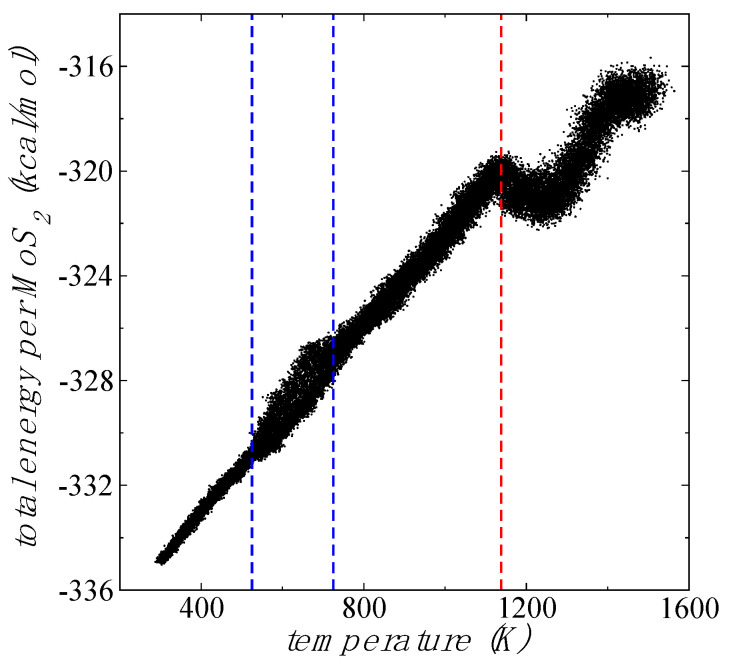
Scan of E_#MoS2_ of 1L Mo_243_S_486_ flake (black points) as a function of the instant flake T, under vacuum and NVT conditions with T increased from 298.15 to 1500 K at a rate of 100 K/300 ps. The discontinuous blue delineates the region of high fluctuations between 525 and 725 K. The discontinuous red line delineates the region of linear dependency of E_#MoS2_ with the T.

A scan of E_#MoS2_ as a function of T between 298.15 and 1500 K is shown in Figure 5. E_#MoS2_decreased with T and indicated that the flake became less unstable as T increases. The change in E_#MoS2_ is interestingly less than 1 eV from 298.15 to 1138 K, which indicated that probably chemical changes did not occur (bond breaking or rearrangement) during this heating process, as seen in the animation (Video S2) in the Supplementary Material. Linear behavior is observed for E_#MoS2_ from 298.15 to 1138 K, which is close to the limit of thermal stability in small flakes of MoS_2_ [51]. Beyond 1138 K, E_#MoS2_ decreased when T was increased. In this region, the system stopped its bending behavior (Video S2), recovered the in-plane vibrations behavior, and some S dimers from the borders started to separate from the flake (Video S2). Around 1258 to 1500 K, the system started again to reduce its stability as T increased. From 525 to 725 K, the system showed a region of large fluctuations for E_#MoS2_, not present at lower or larger T (up to 1138 K), where the fluctuations of E_#MoS2_ were less pronounced. The bending behavior started at lower temperatures (300–400 K), and the high fluctuations zone started at a larger T (525 K). The region of high fluctuations (525–725 K) may be a region where structural changes can be facilitated due to the instability of the system, potentially assisting the transition to the 1T polytype [52].

### 3.2. 2L–2H Flakes

**Figure 6:**
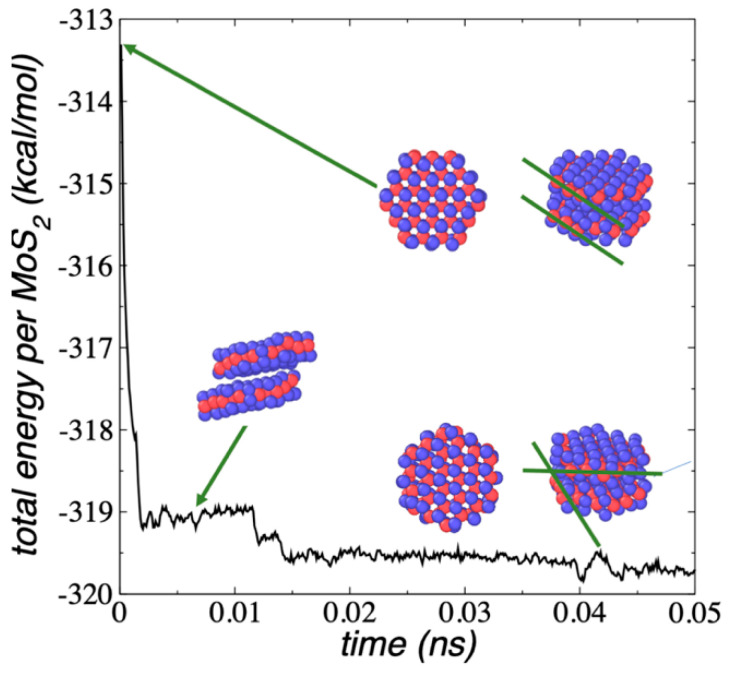
E_#MoS2_ of the Mo_27_S_54_, 2L flake as a function of simulation time, under vacuum and NVT conditions at 298.15 K. The inset snapshots correspond to the initial (same orientation), intermediate (slid) and equilibrated (shifted orientation, 60°) conformations of the flakes. Blue and red spheres represent Mo and S atoms, respectively.

We also studied the effect of mechanical support and analyzed the stability of 2L MoS2 flakes. We tested the most stable orientation between two 1L Mo_27_S_54_, which had the conformation of an equilibrated 1L flake in vacuum at 298.15 K at the beginning of the simulation. We replicated the layer in the normal direction to the surface of the flake (same orientation) and simulated the system under NVT conditions. At the beginning (0.02 ns), the system shows strong repulsions between the layers, and one of the layers is displaced laterally to an intermediate conformation (Figure 6). The laterally displaced conformation is more suitable (less contact area) for transformation, and one of the layers rotated 60° to the conformation of a 2H polytype. The intermediate conformation kept the opposite orientation of the 3R polytype, which lasted for 0.01 ns. The difference between the 3R-like orientation (metastable, intermediate conformation) and the 2H conformation (final conformation, equilibrated) is very small (less than 0.5 kcal/mol), in agreement with the DFT calculations by Yan et al., who found very little differences between the energies of both polytypes [53]. Yan et al. [53] used the projector augmented-wave (PAW) approach, the generalized gradient approximation (GGA) of Perdew-Burke-Ernzerhof (PBE) and the correction scheme of Grimme for interlayer van der Waals interactions. The E_#MoS2_ in the 2L flake (−319.75 kcal/mol) is 1.13 kcal/mol more stable than the E_#MoS2_ of the corresponding 1L flake. We conducted several simulations with different initial conditions and all of them resulted in conformations with rotated orientations (60°) between the layers.

**Figure 7:**
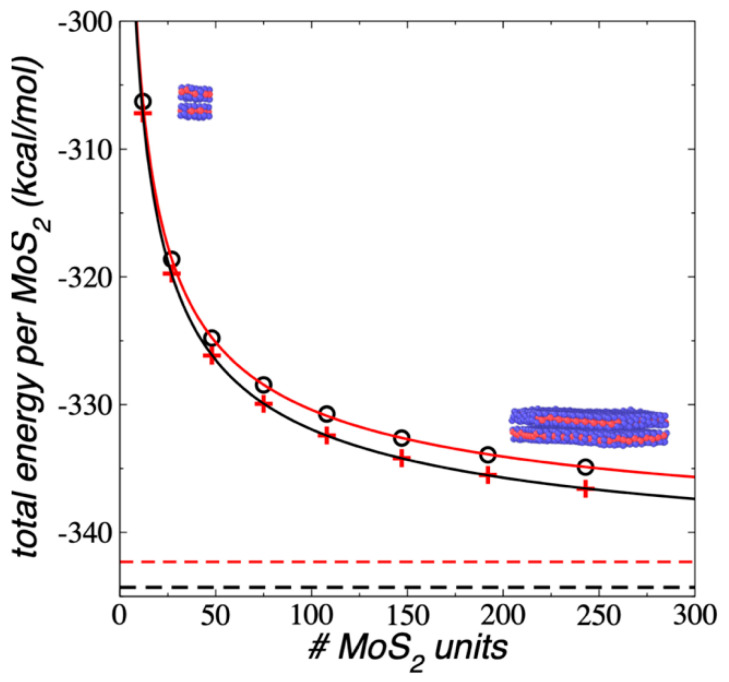
E_#MoS2_ of 2L flakes (red crosses) as a function of the number of MoS_2_ units, under vacuum and NVT conditions at 298.15 K. The black line represents a fitting to an exponential decay expression with the discontinuous black line represent the plateau value. Black circles, and red lines represent the values and fittings for 1L flakes reported in Figure 2. The inset snapshots correspond to the equilibrated conformations of the smallest (2–Mo_12_S_24_) and largest (2–Mo_243_S_486_) flakes. Blue and red spheres represent Mo and S atoms, respectively.

To expedite the calculations, the rest of the layers with smaller and larger areas were simulated using conformations with rotated orientations of 60° between the layers. The E_#MoS2_ results are summarized in Figure 7 as a function of the number of MoS_2_ units in the systems. All systems were equilibrated at NVT conditions and 298.15 K. The behavior is like the one observed in 1L flakes, where the values of E_#MoS2_ exponentially grew with the number of MoS_2_ units, ending in a plateau value more stable than the one obtained for 1L flakes. As the area of the layers grows, E_#MoS2_ slightly grew from the value of 1L flakes, but it never reached differences of more than 1 kcal/mol in this work. The additional stability is due to the contact area created between the two layers. At 298.15 K none of the flakes showed any bending behavior. For very large areas, the difference between 1L and 2L flakes showed a E_#MoS2_ difference of almost 2 kcal/mol.

### 3.3. ML–2H, 3R and Mixed Flakes

**Figure 8:**
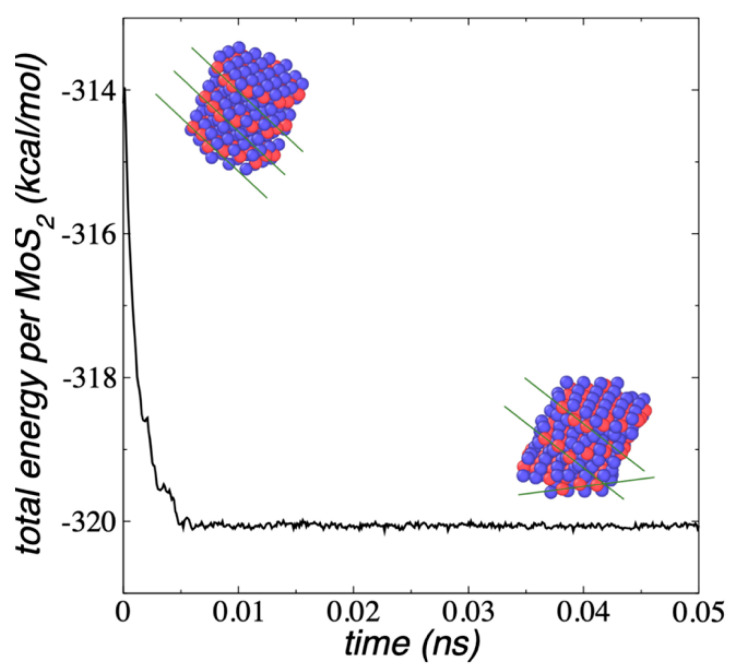
E_#MoS2_ of 3L Mo_27_S_54_ flake as a function of simulation time, under vacuum and NVT conditions at 298.15 K. The inset snapshots correspond to the initial (3R, same orientation in all layers, top) and equilibrated (1-1L with shifted orientation of 60°, bottom) conformations of the flakes. Blue and red spheres represent Mo and S atoms, respectively.

MoS_2_ flakes with three (3L) or more layers were simulated and stabilized under NVT conditions at 298.15 K. We studied the dependence of the stability on the number of layers in the flakes using very small areas (ML flakes with large areas are computationally challenging). We first tested if systems with 3L are constrained enough to avoid the 3R to 2H transformation observed in 2L flakes in the previous section, which involved the rotation of one of the layers. We constructed 3L Mo_27_S_54_ flakes with the same orientation (replicated from an equilibrated 1L flake at the same T) as that observed in a 3R polytype. The system equilibrated after 0.005 ns (Figure 8) under NVT conditions at 298.15 K. The equilibration involved, as in the 2L flake, a coupled displacement and reorientation of one of the layers, while the rest of the layers maintained their 3R orientation. The final conformation involved a mixed polytype flake. Similar behavior has been observed using DFT calculations in the work of Yan et al., who found that mixed 2H/3R polytypes are stable, but pure polytypes are more stable [53].

**Figure 9:**
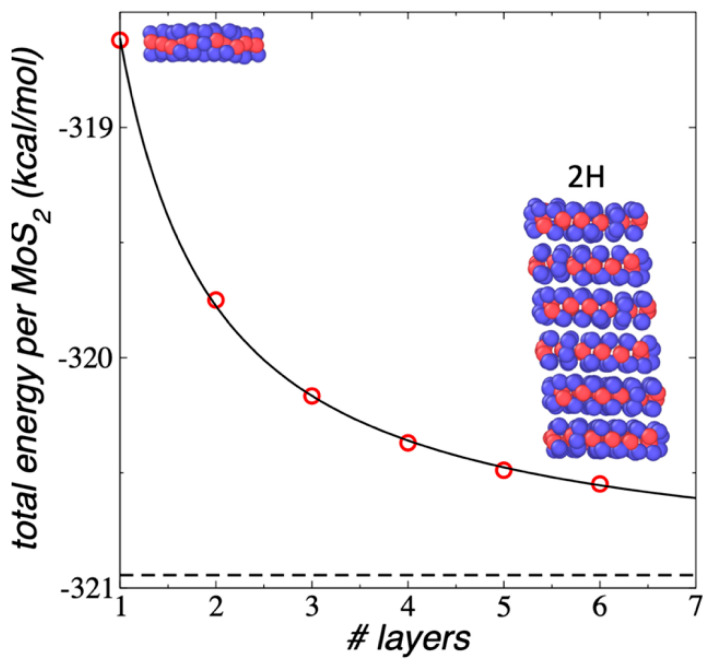
E_#MoS2_ of 1L, 2L, and ML Mo_27_S_54_ flakes (red circles) as a function of the number of MoS_2_ units, under vacuum and NVT conditions at 298.15 K. The black line represents a fitting to an exponential decay expression with the discontinuous black line represent the plateau value. The inset snapshots correspond to the equilibrated conformations of the 1L (1–Mo_27_S_54_) and ML (6–Mo_27_S_54_) flakes. Blue and red spheres represent Mo and S atoms, respectively.

We studied the dependency of the number of layers on the stability of the ML flake using flakes with small areas (Mo_27_S_54_). The stability results for 1L, 2L, and flakes up to 6L are reported in Figure 9 as a function of the number of layers contained in the system. The flakes were studied under NVT conditions at 298.15 K. The layers of all systems had orientations corresponding to 2H polytypes, which did not change during the simulations. In the equilibrated conformations, the flakes showed a small tilting angle along the normal direction to the surfaces of the layers. Like the effect of the size of the area of the flake, an increasing number of layers make the flake more stable, and the results also fit well to an exponential decay function. Compared to a 1L flake, the stability gained in ML flakes with many layers is small (2.3 kcal/mol), effectively achieved in ML flakes with a relatively small number of layers (25), which showed a difference with the plateau value of only 0.1 kcal/mol. It is expected that larger stability will be gained in ML flakes with larger areas.

### 3.4. Ring-like Flakes

**Figure 10:**
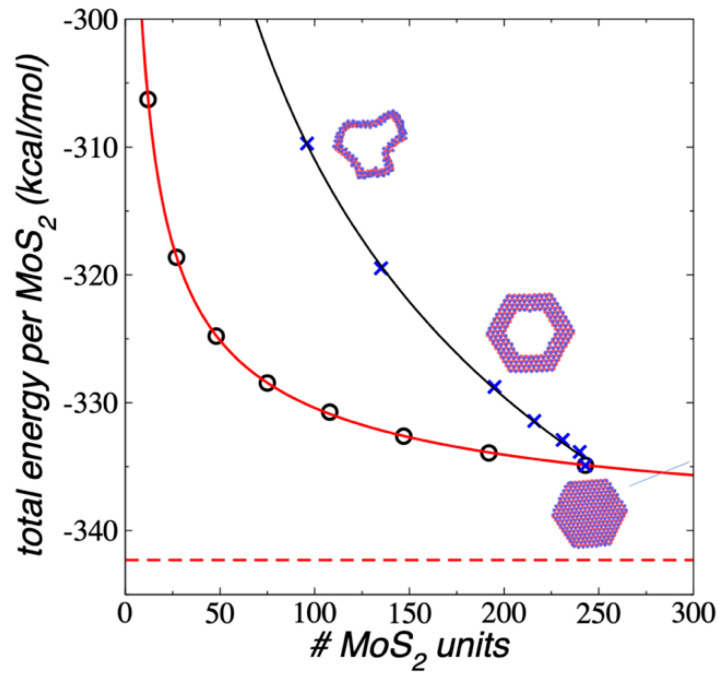
E_#MoS2_ of ring-like 1L flakes (blue crosses) as a function of the number of MoS_2_ units, under vacuum and NVT conditions at 298.15 K. The black line represents a fitting to a power function on the total number of MoS_2_ units. Black circles, and red lines represent the values and fittings of 1L flakes reported in Figure 2. The inset snapshots corresponded to the equilibrated conformations of the ring-like 1L Mo_96_S_192_ flake (top), Mo_147_S_294_ flake (center), and the full-covered flake Mo_243_S_486_ (bottom). Blue and red spheres represent Mo and S atoms, respectively.

We tested the stability of 1L flakes with holes, which can be created experimentally using electron beams [54,55,56] or the impact of energetic nanoparticles [40], and these porous flakes are the basis of the proposed layered MoS_2_ nanotubes presented later in this paper. For the 1L Mo_243_S_486_ flake under NVT conditions at 298.15 K, we removed central areas containing rings with 3, 12, 27, 48, 108, and 147 MoS_2_ units in total, maintaining the outer diameter and allowing the system to stabilize, creating porous (ring-like) flakes that were more flexible than the full-covered layers. The created ring-like flakes contained between two and seven concentric rings of MoS_2_ units. We were not able to simulate flakes with only one concentric ring, as previously observed in STEM images of MoS_2_ nanowires, which, at minimum, contained 2 MoS_2_ units [56]. These flakes were stable for up to several ns of simulation. Figure 10 summarizes the results of E_#MoS2_ as a function of the number of MoS_2_ units and compared the results to the full-covered flakes. The values showed that the E_#MoS2_ decreased with the total number of MoS_2_ units, but it did not fit to an exponential decay function. Instead, it was fitted well to a power function based on the total number of MoS_2_ units. The cohesiveness of these ring-like flakes was lower than the cohesiveness of the full-covered layers with the same total number of MoS_2_ units, but similar when we compared them in terms of the number of rings that form the flakes. For example, the smallest full-covered flake studied in this work contained two concentric rings of MoS_2_ units (a central ring with three MoS_2_ units and the border ring containing nine MoS_2_ units) and E_#MoS2_ of −306.28 kcal/mol, while the thinnest ring-like flake studied in this work, Mo_243_S_486_–Mo_147_S_294_ (Mo_96_S_192_), also consisted of two concentric rings (containing 51 and 45 MoS_2_ units) and had an E_#MoS2_ value of −309.72 kcal/mol (a difference of 3.44 kcal/mol in each MoS_2_ unit). The difference reduced when we compared ring-like flakes with smaller holes; the difference between the full-covered flake Mo_147_S_294_ and the ring-like flake Mo_243_S_486_–Mo_3_S_6_ (Mo_240_S_480_), which both contained seven rings, was only 1.23 kcal/mol.

### 3.5. ML–2H and 3R Nanotubes

**Figure 11:**
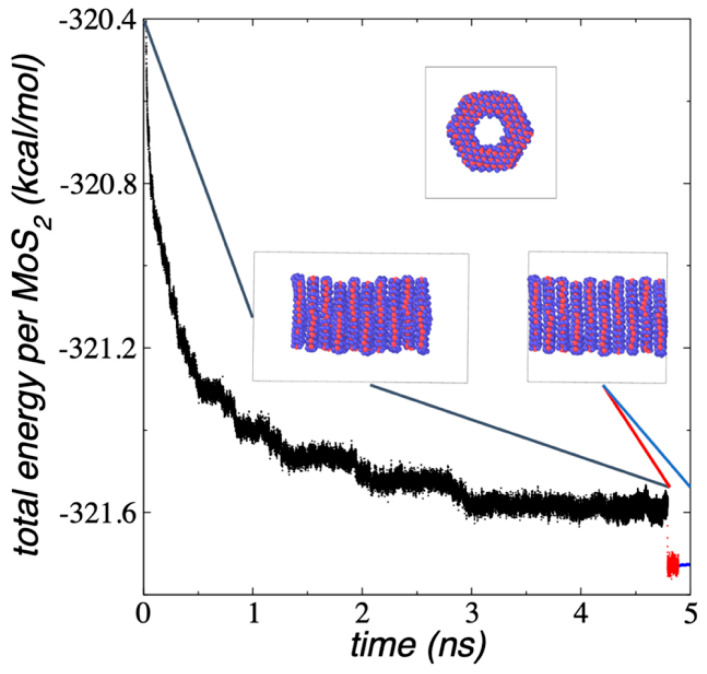
E_#MoS2_ of the Mo_108_S_216_-Mo_27_S_54_, 10L nanotube with 2H orientation as a function of the simulation time, under vacuum and NVT conditions at 298.15 K (black points), as a periodic nanotube at NVT conditions at 298.15 K (red points) and as a periodic nanotube under NVE conditions (blue points). The inset snapshots correspond to normal (top) and lateral (bottoms) views of the nanotube. Black lines in the bottom snapshots correspond to the actual limits of the periodic simulation cells. Blue and red spheres represent Mo and S atoms, respectively.

Nanotubes made of multiple 1L porous MoS_2_ using the 2H orientation between the layers (Figure 11) are held together through van der Waals interactions between the sulfur atoms of the multiple surface layers. The nanotube was built from the configuration of a 1L Mo_108_S_216_ and erasing the atoms in the center of the layer, corresponding to a 1L Mo_27_S_54_ flake (Mo_108_S_216_-Mo_27_S_54_). The conformation was replicated 10 times (10L) at interlayer separations of 6.4 Å and the orientation between adjacent layers were rotated by 60°. The ML nanotube was simulated within a simulation cell large enough to be considered in vacuum under NVT conditions at 298.15 K. Figure 11 shows a lateral view of the nanotube and the pore developed inside the layers. The proposed initial conformation was structurally very similar to the conformation corresponding to the minimized energy, and the layers only slightly moved in the normal direction to the surface layers. After 3 ns of simulation (Figure 11), the systems stabilized with an E_#MoS2_ of −321.59 kcal/mol per MoS_2_ unit, in which every MoS_2_ unit is on average 20.71 kcal/mol less cohesive than the E_#MoS2_ estimated for infinite surfaces of 1L flakes. The stability may be decreased due to the inner edges and the small area of the ring-like flakes used to build the nanotube, while stability may be slightly increased due to the number of interacting surfaces of the flakes in the nanotube.

In equilibrium, the average separation between layers remained at 6.4 Å, and we used this separation to simulate an infinite nanotube by reducing the dimension of the simulation cell in the normal direction to the exact value of 64 Å (10 layer–layer separations). The layer separation obtained in this work is similar to that observed in experiments with ML MoS_2_ (6.5 Å) [57] and commercial MoS_2_ (6.2 Å) [58], which has been attributed to strong sulfur–sulfur interlayer interactions. Controlling the synthesis T, different polytypes arise experimentally, and the interlayer separation can reach values in the range of 6.2 to 6.8 Å [59]. We attempted to optimize this separation by increasing the size of the simulation cell in the normal direction, but this only resulted in less cohesive systems, and size reductions resulted in conformational changes, which could lead to a layer expulsion after long periods of simulation time. The adjusted simulation cell allowed us to mimic a continuous nanotube with a slightly larger E_#MoS2_ (−321.79 kcal/mol), which is due to the additional contact between the end layers, and it maintained its conformational geometry and T, even when the system was simulated under NVE conditions.

**Figure 12:**
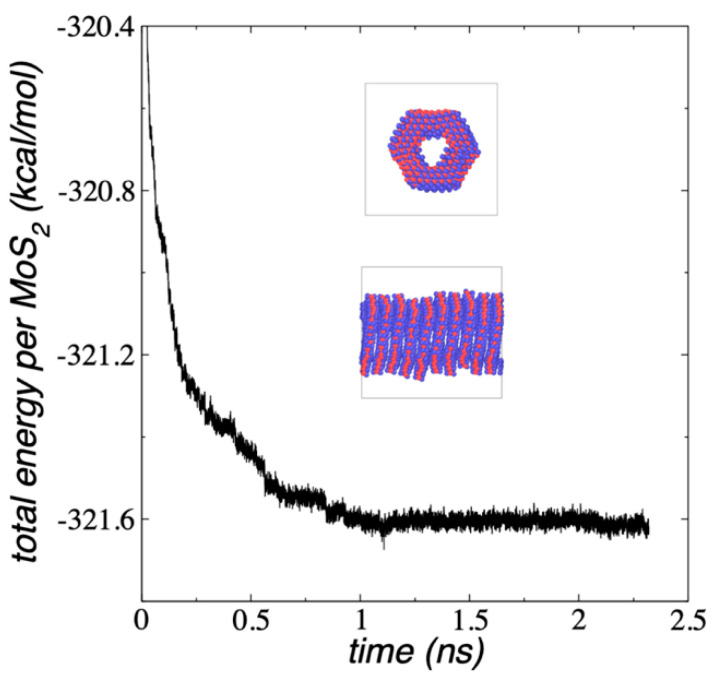
E_#MoS2_ of the Mo_108_S_216_-Mo_27_S_54_, 10L nanotube with the 3R orientation as a function of the simulation time, under periodic simulation cell and NVT conditions at 298.15 K. The inset snapshots correspond to normal (top) and lateral (bottoms) views of the nanotube. Black lines in the bottom snapshot correspond to the actual limits of the periodic simulation cell. Blue and red spheres represent Mo and S atoms, respectively.

We also simulated nanotubes using the 3R conformation, where all the layers had the same orientation (Figure 12). This nanotube was also built from the configuration of a single Mo_108_S_216_-Mo_27_S_54_ flake, repeated 10 times in the normal direction with interlayer separations of 6.4 Å. The nanotube was simulated only as a periodic tube to avoid the problem of generating mixed 2H/3R conformations. The system equilibrated faster than the non-periodic 2H conformation and required only 1 ns to stabilize, probably due to the spatial restrictions of the simulation cell. The periodic 3R nanotube is slightly less stable than the corresponding periodic 2H nanotube (+0.18 kcal/mol per MoS_2_ unit), similar to the results previously obtained with 3L flakes using DFT calculations by Yan et al. [53]. 

Snapshots of the 2H and 3R MoS_2_ nanotubes showed that the layers of the 2H nanotube are more ordered than the 3R nanotube, which generated a free space inside the nanotube that is more open than the 3R nanotube. The more open 2H nanotube will adsorb and conduct larger species than the 3R nanotube.

## 4. Conclusions

Molecular dynamics simulations using a reactive potential showed that non-concentric, ML nanotubes made of 1L porous MoS_2_ flakes and inner/outer diameters of 1.35/3.04 nm are energetically stable under NVT conditions at 298.15 K. Periodic 10L nanotubes with 2H and 3R orientations showed very similar total energies, comparable to the energy minimization results reported by Yan et al. [53] for systems with a very small number of atoms and up to 3L, which were estimated through DFT calculations at 0 K. Compared to 2L flakes with macroscopic areas, each MoS2 unit in the periodic nanotubes are 1 eV less stable on average, which is due to two main effects: small number of concentric rings that form each porous 1L, and additional contact areas between the layers. The number of concentric rings in porous or full-covered 1L flakes is directly related to the stability of the flake, and flakes with a small number of rings showed a strongly decreased stability. The creation of areas of contact between the layers has a minor effect (increasing stability), and it is less important in ring-like flakes, which had less contact area.

## Data Availability

The data presented in this study are available on request from the corresponding author.

## Supplementary Materials

The following supporting information can be downloaded at: https://www.mdpi.com/article/10.3390/membranes12080818/s1, Video S1: Bending behavior of Mo_243_S_486_ under NVT conditions at 1100 K. Video S2: Heating of Mo_243_S_486_ under NVT conditions from 1100 to 1500 K.
